# Design and Application of Thymol Electrochemical Sensor Based on the PtNPs-CPOFs-MWCNTs Composite

**DOI:** 10.3390/molecules28083398

**Published:** 2023-04-12

**Authors:** Na Li, Hongyue Zhang, Min Cui, Jujie Ren, Jingru Huang, Bao Sun, Haiyan Zhao, Cong Zhang

**Affiliations:** Hebei Provincial Key Laboratory of Photoelectric Control on Surface and Interface, School of Sciences, Hebei University of Science and Technology, Shijiazhuang 050018, China

**Keywords:** polyoxometalate, CPOFs, thymol sensor, electrochemistry

## Abstract

In this study, the preparation of covalent polyoxometalate organic frameworks (CPOFs) is introduced using the idea of polyoxometalate and covalent organic frameworks. Firstly, the prepared polyoxometalate was functionalized with an amine group (NH_2_-POM-NH_2_), and then the CPOFs were prepared by a solvothermal Schiff base reaction with NH_2_-POM-NH_2_ and 2,4,6-trihydroxybenzene-1,3,5-tricarbaldehyde (Tp) as monomers. After the incorporation of PtNPs and MWCNTs into the CPOFs material, the PtNPs-CPOFs-MWCNTs nanocomposites, which possess excellent catalytic activity and electrical conductivity, were formed and utilized as new electrode materials for the electrochemical thymol sensors. The obtained PtNPs-CPOFs-MWCNTs composite exhibits excellent activity toward thymol, which is attributable to its large special surface area, good conductivity and the synergistic catalysis of each component. Under optimal experimental conditions, the sensor presented a good electrochemical response to thymol. The sensor shows two good linear relationships between the current and thymol concentration in the range of 2–65 μM (R^2^ = 0.996) and 65–810 μM (R^2^ = 0.997), with the corresponding sensitivity of 72.7 μA mM^−1^ and 30.5 μA mM^−1^, respectively. Additionally, the limit of detection (LOD) was calculated to be 0.2 μM (S/N = 3). At the same time, the prepared thymol electrochemical sensor revealed superior stability and selectivity. The constructed PtNPs-CPOFs-MWCNT electrochemical sensor is the first example of thymol detection.

## 1. Introduction

Terpenoids are important compounds in Chinese herbal medicine. Terpenoids arouse the interest of many scientists because of their several different health properties [1]. Thymol (2-isopropyl-5-methylphenol), a terpenoid compound, is one of the most important phenolic oils in the thymus. It has been reported that thymol has antibacterial, antioxidant and anti-aging properties in mammals and is bactericidal, stronger than phenol and less toxic [2,3]. It is often used as a disinfectant to control varroa mites in the bee industry. Thymol residues in honey, although non-toxic, may affect the quality of honey and lead to changes in its taste [4,5,6]. Additionally, thymol is a common active ingredient used in topical drugs, food flavors, mouthwashes and disinfectants [7,8,9]. In view of the widespread existence of thymol in our life, rapid analytical methods are of great significance in determining thymol content in different samples.

High-performance liquid chromatography (HPLC) [10], gas chromatography-mass spectrometry [11] and spectrophotometry [12] are conventional techniques to detect thymol. In comparison with these methods, the electrochemical method has attracted much attention due to its simple operation, low cost, fast detection, high sensitivity and good selectivity. Thymol has an electrochemical active group, which makes it more suitable for measurement in the detection through the electrochemical method. For example, the Robledo group [13] determined thymol and carvacrol in essential oils by GCE. Aghamohseni’s laboratory [14] used MnY/CPE for the determination of thymol. Modification of the electrode surface can enhance analytical detection and improve sensitivity; therefore, the selection of modification material plays a critical role.

Polyoxometalates (POMs) constitute a class of unique metal oxide cluster compounds and display a variety of advantages, including remarkable redox abilities, modification and easy organic grafting [15,16]. At present, electrochemical sensors based on POMs have been widely used for the detection of Cr^6+^, nitrite, bisphenol A, dopamine, uric acid and L-tryptophan [17,18,19,20,21]. In addition, POMs not only have good development in electrochemistry but also exhibit a broad application prospect in biology, batteries and magnetism. Xu et al. used zeolitic imidazolate framework-8 (ZIF-8) to anchor polyoxometalate (POM) and cooperate with xanthine oxidase (XOD) to achieve multistep cascade amplification of electrochemical signals. In the presence of subxanthine, XOD catalyzes the generation of H_2_O_2_, while POMs catalyze the decomposition of H_2_O_2_ and amplify the electrochemical signal of thionine. The current response of thionine enhances the ultra-sensitive detection of CYFRA21-1. It provides a way for the gentle preparation of probes with high catalytic site dispersion [22]. Han et al. used the conductive polypyrrole (PPy) and nitrogenous ligands (1,10-phenanthroline monohydrate = 1,10-phen) for metal-organic frameworks to fabricate a [Cu(1,10-phen)(H_2_O)_2_]_2_[Mo_6_O_20_]@Ppy (Cu-POMOF@PPy) composite to effectively improve the electrochemical stability. This work not only presents a new preparation method for POM-based lithium-ion batteries but also expands the types of anode materials [23]. Wang et al. reported two Keggin POM-based compounds substituted by Ni clusters, an isolated cluster, and a 2-D framework through the hydrothermal method. The third-order nonlinear optical measurements of compound 1 show that it has a good NLO response and could be a potential nonlinear optical material (β = 0.003384 cm GW^−1^, σ = 2216 GM) [24]. However, POMs have high solubility and low specific surface area, which are very unfavorable; however, when combined with porous frame materials, they can solve the above problems well.

Since Yaghi et al. reported covalent organic frameworks (COFs) in 2005 [25] as an emerging class of porous polymers, they have been widely used in catalysis, gas adsorption, energy storage, etc., because of their excellent chemical properties. Hui Zhao used an etched halloysite nanotube as a material on the sensing platform and the COF/MnO_2_ nanocomposite for signal amplification; an electrochemical biosensor was constructed for detecting human chorionic gonadotropin. This work developed an ultrasensitive assay for the electrochemical detection of human chorionic gonadotropin [26]. Signal-on electrochemical aptasensors were fabricated for the sensitive and label-free determination of cardiac troponin I by Feng Gao [27]; the biosensors showed a low detection limit of 2.6 fg mL^−1^ with a linear range from 10 fg mL^−1^ to 10 ng mL^−1^ for cardiac troponin I. However, the preparation of COFs was mainly limited to aromatic organic units until now. In theory, by combining modified POMs with appropriate organic building blocks, covalent polyoxometalate-organic frameworks (CPOFs) can be constructed, and the injection of inorganic components makes CPOFs have richer functional and structural diversity [28]. Recently, CPOFs constructed from inorganic and organic building blocks were reported and applied to battery and dye degradation, respectively, with good results. Fang et al. reported three-dimensional porous crystalline CPOFs that displayed high reversible capacity [29]. Yang et al. reported the three isostructural complexes M-Anderson-COFs with a three-interpenetrated diamondoid network. Among them, Mn Anderson-COF exhibited the highest catalytic activity in the photodegradation of RhB and MB in water [30]. As far as we know, there are no reports on the electrochemical sensing of CPOFs, but it is necessary to develop CPOF-based nanomaterials with high conductivity and excellent catalytic activity for the construction of electrochemical sensors because POMs and COFs both have poor conductivity. It has been reported that functional metal nanoparticles (MNPs) could be loaded onto the surface of COFs to improve the active site [31]. At the same time, due to the excellent conductivity and stability of carbon nanomaterials, multi-walled carbon nanotubes (MWCNTs) were further introduced to enhance the catalytic activity [32]. The electrochemical sensor has the advantages of high sensitivity and low detection limit [33,34]. This inspired us to construct the MNPs-CPOFs nanocomposites with carbon nanomaterials for the detection of thymol.

**Scheme 1:**
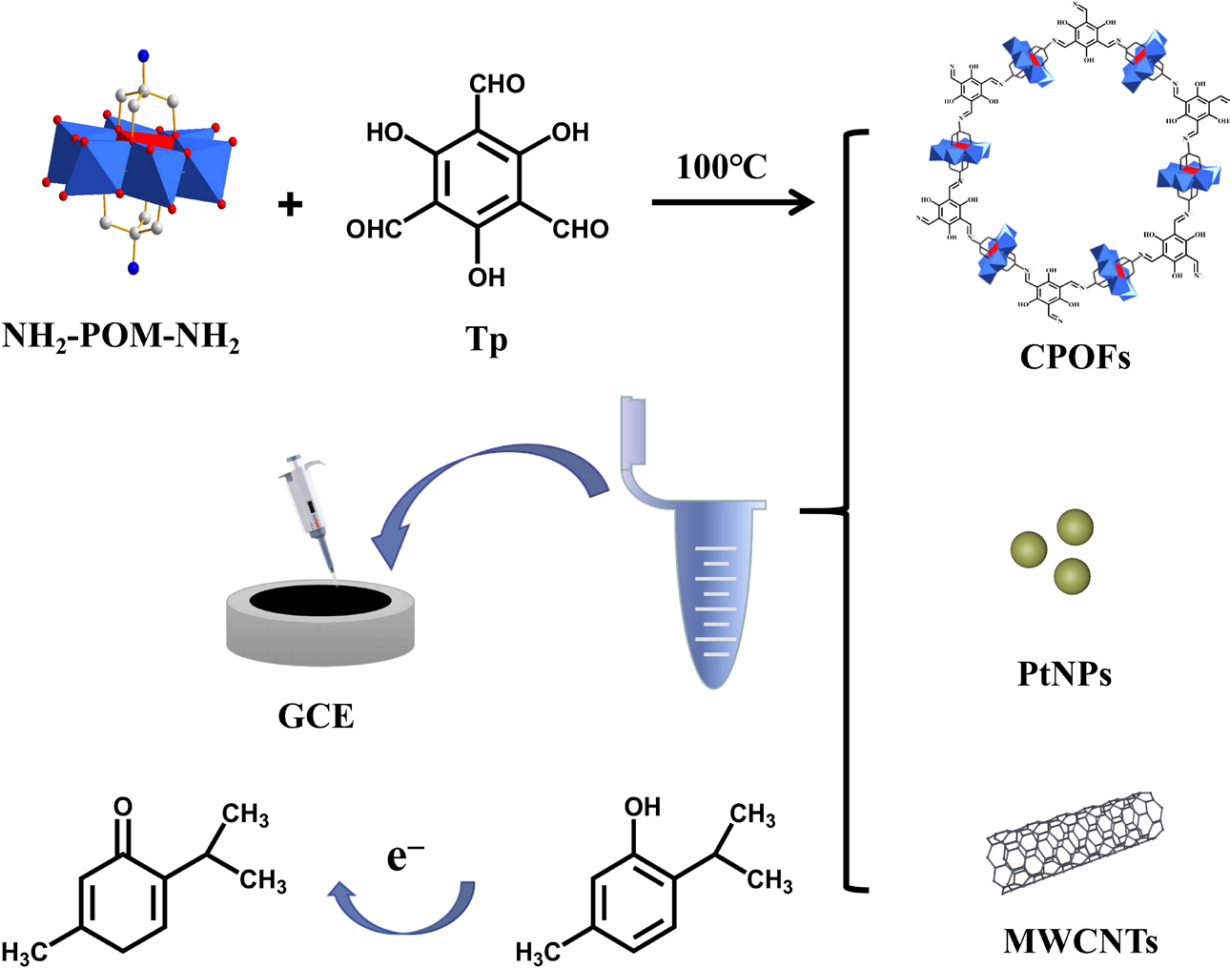
The schematic diagram for the fabrication of the thymol sensor.

In the present study, a sensitive electrochemical sensor for thymol detection was constructed based on CPOFs decorated with PtNPs and MWCNTs (PtNPs-CPOFs-MWCNTs) as electrode materials. As shown in Scheme 1, we successfully obtained the CPOFs by connecting modified inorganic clusters with organic ligands. Then, PtNPs and MWCNTs were loaded onto the surface of CPOFs to enhance the electrical conductivity and catalytic performance. As expected, the composite-modified electrode realized the quantitative detection of thymol. The constructed sensor opened a new direction for the application of CPOFs in the field of drug detection.

## 2. Results and Discussion

### 2.1. Characterizations of the Composites

**Figure 1:**
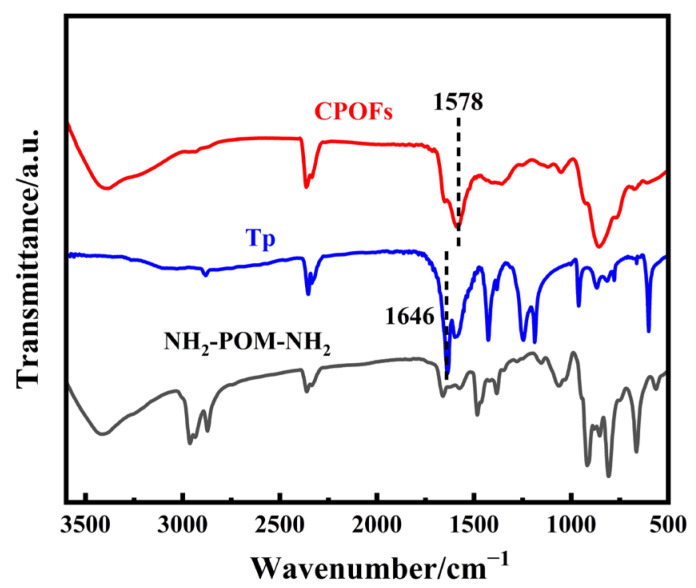
FT-IR spectra of NH_2_-POM-NH_2_, Tp and CPOFs.

The chemical structure of the nanocomposites was then confirmed by FTIR spectroscopy (Figure 1). The existence of the characteristic peaks at 923, 853 and 807 cm^−1^ were stretching vibrations of Mo=O, and the peaks at 662 and 561 cm^−1^ corresponded to the stretching vibration of O-Mo-O [35]. The –NH stretching bands of NH_2_-POM-NH_2_ (3100–3400 cm^−1^) may be obscured by water, and the C=O stretching bands of Tp (1646 cm^−1^) disappeared in the spectrum of CPOFs. The existence of the characteristic peaks at 1578 cm^−1^ (C=N) revealed that a condensation reaction had occurred, which was followed by the successful fabrication of CPOFs [36].

**Figure 2:**
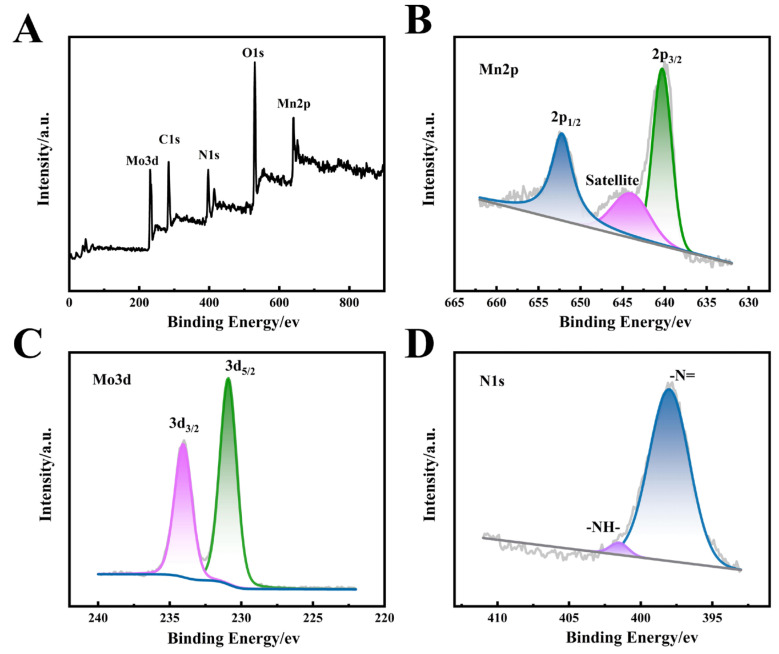
XPS full-spectrum analysis of CPOFs (**A**); Mn 2p region in XPS of CPOFs materials (**B**); Mo 3d region (**C**); N 1s region (**D**).

X-ray photoelectron spectroscopy (XPS) was used to analyze the chemical composition of CPOFs nanomaterials. First, Figure 2A shows the XPS full-spectrum analysis of CPOFs nanomaterials, which clearly exhibited the presence of C 1s, N 1s, O 1s, Mo 3d and Mn 2p elements in CPOFs nanomaterials. As shown in Figure 2B, the typical peaks of the Mo 3d_5/2_ and Mo 3d_3/2_ were about 230.91 eV and 234.06 eV, respectively [21], which proved the existence of Mo element in CPOFs nanomaterials. The narrow-spectrum analysis of Mn 2p is shown in Figure 2C. Two main peaks were shown in the XPS spectrum, which were located at 640.28 eV and 652.22 eV corresponding to Mn 2p_3/2_ and Mn 2p_1/2_, respectively. The small peak at 643.98 eV was for the satellite peak of Mn 2p_3/2_ [37]. The N 1 s spectra peak appeared at 398.01 eV and 401.5 eV (as shown in Figure 2D) belonging to the unsaturated C = N of CPOFs keys and residual -NH_2_ groups, respectively [38]. The above results confirmed that CPOFs nanomaterials were successfully prepared by NH_2_-POM-NH_2_ and Tp monomer.

**Figure 3:**
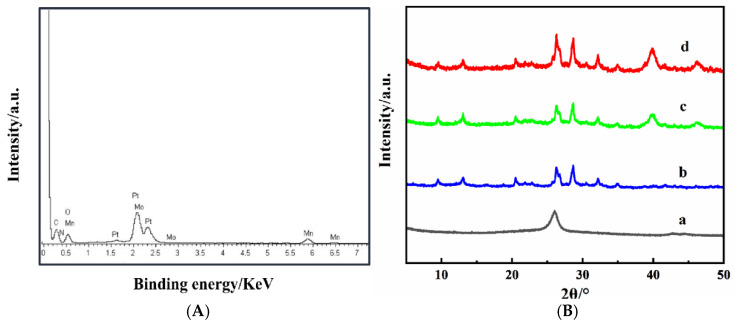
(**A**) EDS diagram of PtNPs-CPOFs; (**B**) MWCNTs (a); CPOFs (b); PtNPs-CPOFs (c); XRD pattern of PtNPs-CPOFs-MWCNTs (d).

The formation of the PtNPs-CPOFs composites was confirmed by EDS. EDS of the PtNPs-CPOFs nanocomposite is illustrated in Figure 3A, and the content of the elements of the PtNPs-CPOFs nanocomposite is given in Table S1. The PtNPs-CPOFs nanocomposite was composed of carbon, nitrogen, oxygen, manganese, molybdenum and platinum. The PtNPs-CPOFs nanocomposites have been successfully prepared. Figure 3B shows the XRD patterns of MWCNTs, CPOFs, PtNPs-CPOFs, and PtNPs-CPOFs-MWCNTs. It can be seen from the figure that after the introduction of PtNPs, clear visible peaks appeared at 39.88° and 46.08°, which correspond to the crystal planes (1 1 1) and (2 0 0) of PtNPs, respectively, which is consistent with the literature. No peak of MWCNTs was found after the addition of MWCNTs, which may be because the diffraction peak of MWCNTs at 26.02° overlapped with that of the original CPOFs.

**Figure 4:**
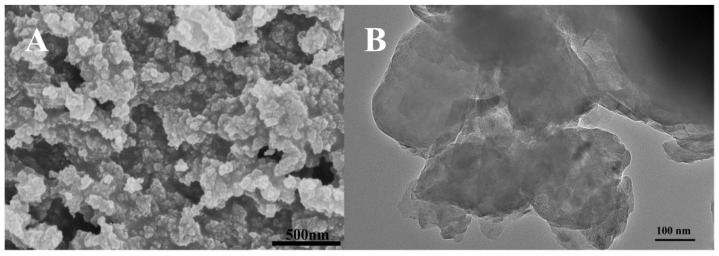
(**A**) SEM image of CPOFs; (**B**) TEM image of CPOFs.

**Figure 5:**
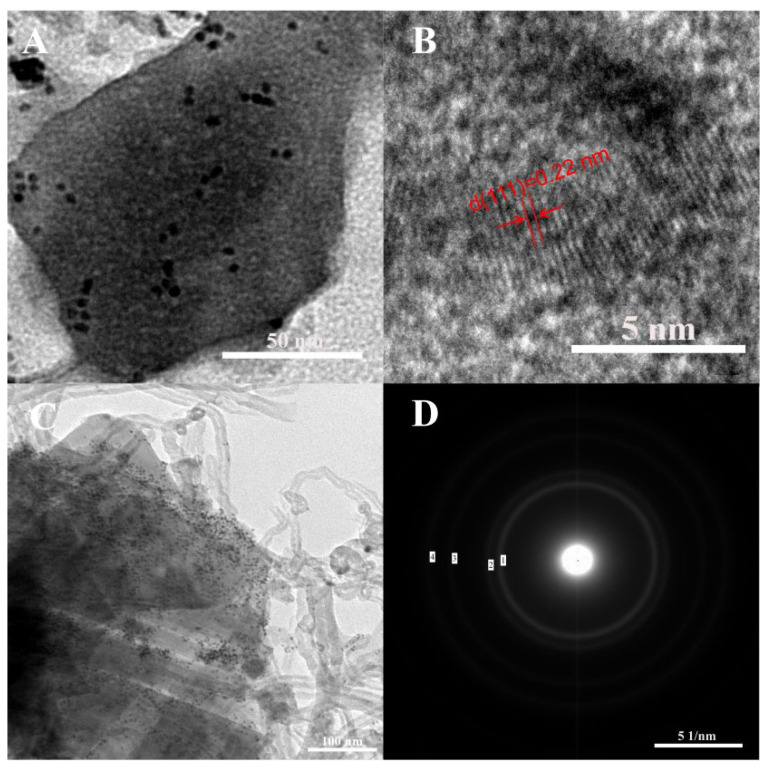
(**A**) TEM image of PtNPs-CPOFs; (**B**) HRTEM diagram of PtNPs-CPOFs; (**C**) TEM images of PtNPs-CPOFs-MWCNTs; (**D**) Electron diffraction pattern of PtNPs.

In order to observe the morphology of the prepared materials, SEM and TEM were performed. Figure 4A,B show the SEM and TEM images of CPOFs, respectively. It can be seen from the morphological characterization that the prepared CPOFs have an irregular shape. The PtNPs-CPOF and PtNPs-CPOFs-MWCNT composites were characterized by TEM experiments, and the results are shown in Figure 5. PtNPs were dispersed on the surface of CPOFs (Figure 5A), and MWCNTs were long tubular (Figure 5C). Under high-resolution TEM images, most of the synthesized PtNPs were 3.92 nm in size, and the spacing of adjacent lattice fringes was 0.22 nm, corresponding to the (1 1 1) crystal plane of PtNPs (Figure 5B). Electron diffraction was performed on PtNPs (Figure 5D), and the appearance of diffusion rings in the electron diffraction mode indicated that polycrystalline PtNPs had been formed.

To illustrate the thermal stability of the materials, the TG of CPOFs, PtNPs-CPOFs and PtNPs-CPOFs-MWCNTs were tested with a heating rate of 10 °C/min from room temperature to 1000 °C under a nitrogen atmosphere (Figure S1). The results indicate that these materials have high thermal stability.

### 2.2. Electrochemical Characterization of Modified Electrodes

**Figure 6:**
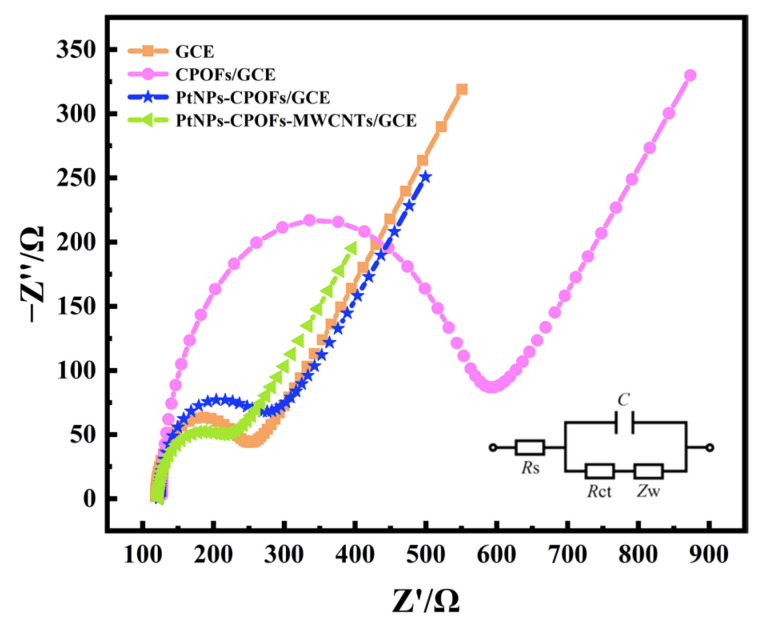
EIS of GCE, CPOFs/GCE, PtNPs-CPOFs/GCE and PtNPs-CPOFs-MWCNTs/GCE in 0.1 M KCl solution containing 5.0 mM [Fe(CN)_6_]^3−/4−^ solution.

Electrochemical impedance spectroscopy (EIS) was used to study the electrical conductivity of the electrode materials and monitor the modification process of the electrodes. The experiment was carried out in a solution containing 5.0 mM [Fe(CN)_6_]^3−/4−^ and 0.1 M KCl. The electrical equivalent circuit models using the ZSimpWin software version 1.0.0.0 were employed to simulate the impedance results. As shown in Figure 6, the resistance values revealed the following trend: CPOFs/GCE > PtNPs-CPOFs/GCE > bare GCE > PtNPs-CPOFs-MWCNTs/GCE. The Rct of the bare GCE was about 116.2 Ω. While after modifying the GCE with CPOFs, the Rct increased to 420.1 Ω mainly because of the intrinsically low conductivity of CPOFs. Compared with the CPOFs/GCE, the Rct of the PtNPs-CPOFs/GCE decreased to 133.2 Ω, which further decreased to 85.4 Ω when MWCNTs were introduced to the PtNPs-CPOFs composites, suggesting that the electrical conductivity of CPOFs was significantly improved by the incorporation of PtNPs and MWCNTs. The above results have confirmed the successful construction of the PtNPs-CPOFs-MWCNT nanocomposite and its excellent electrochemical performance.

**Figure 7:**
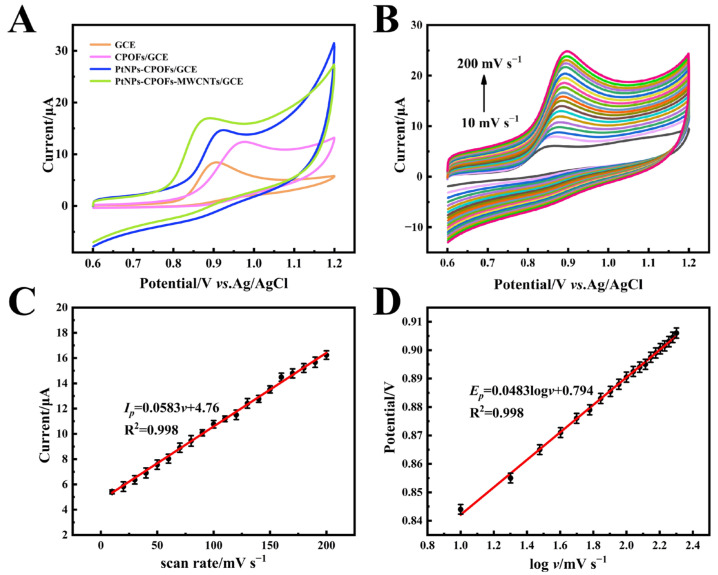
(**A**) CV of bare GCE, CPOFs/GCE, PtNPs-CPOFs/GCE and PtNPs-CPOFs-MWCNTs/GCE in 0.1 M H_2_SO_4_ with 0.2 mM thymol (Scan rate: 100 mV s^−1^); (**B**) CVs of the PtNPs-CPOFs-MWCNTs/GCE in 0.1 M H_2_SO_4_ with 0.2 mM thymol at a different scan rate (from 10 to 200 mV s^−1^); (**C**) The linear relationship of anodic peak current values against the scan rates; (**D**) Plot of the oxidative peak potential versus the logarithm of the scan rate.

Having demonstrated the successful synthesis of PtNPs-CPOFs-MWCNTs, we investigated the electrochemical behavior of these different modified electrodes toward thymol analysis. The electrocatalytic responses of thymol were investigated via CV in 0.1 M H_2_SO_4_ in the presence of 0.2 mM thymol at a scan rate of 100 mV s^−1^. As shown in Figure 7A, an oxidation peak of thymol was observed at each electrode. However, compared with other electrodes, the PtNPs-CPOFs-MWCNTs/GCE with the highest electrical conductivity possessed a higher oxidation peak current and more negative peak potential, which reduced the difficulty of the oxidation reaction. The enhanced electrocatalytic activity arises from the synergistic effect among CPOFs with a large specific surface area and extra high porosity, PtNPs with catalytic activity and favorable conductivity and MWCNTs with superior electrical conductivity.

### 2.3. Optimization of Experimental Conditions

In order to promote the sensitivity of the constructed electrochemical thymol sensor, the experimental conditions were optimized. Firstly, the influence of the amount of PtNPs in the PtNPs-CPOFs-MWCNTs composite on the electrochemical response of thymol was investigated. As shown in Figure S1, when the volume ratio of PtNPs to CPOFs was less than 5:1, the oxidation peak current of thymol increased with the increase in the volume of PtNPs. However, when the volume ratio of PtNPs to CPOFs was greater than 5:1, the peak current of thymol decreased. The increase in the peak current was due to the increase in PtNPs on the electrode, which provided more electroactive sites for thymol oxidation. By contrast, a higher amount of PtNPs would likely block electron transfer, resulting in a lower peak current. Therefore, we chose 5:1 as the volume ratio of PtNPs to CPOFs to prepare PtNPs-CPOFs, which were further used for binding MWCNTs.

The effect of the volume ratio of PtNPs-CPOFs and MWCNTs on the electrocatalytic reaction of thymol was also investigated. As shown in Figure S2, the peak current increased with the increase in MWCNT content in the nanocomposites. However, when the volume ratio of MWCNTs to PtNPs-CPOFs was greater than 5:4, the surface-coated MWCNTs blocked the catalytic site, and the peak current decreased. Therefore, the volume ratio of MWCNTs to PtNPs-CPOFs can be selected as 5:4 to prepare PtNPs-CPOFs-MWCNTs nanomaterials.

Furthermore, the suspension volume of PtNPs-CPOFs-MWCNTs had a significant effect on the oxidation current of thymol. As presented in Figure S3, with an increase in the dropping volume of the PtNPs-CPOFs-MWCNTs composite from 2 μL to 12 μL, the current response first increased, then reduced when the dropping volume exceeded 6 μL. The reason for this change might be attributed to the thicker film on the electrode surface, which prevents the transfer of electrons. Therefore, 6 μL was used as the best dropping volume for the later experiments.

Several supporting electrolyte solutions, such as B-R, H_2_SO_4_, NaOH, citrate-sodium citrate (CA) and PBS (all with 0.1 M concentration), were used as the supporting electrolytes for the DPV technique quantification of thymol (Figure S4). It can be seen that a higher oxidation peak current was observed in H_2_SO_4_ than in other buffers. Therefore, H_2_SO_4_ was selected as the supporting electrolyte.

The effect of the scan rate at the PtNPs-CPOFs-MWCNTs/GCE was also studied by CV in 0.1 M H_2_SO_4_ containing 0.2 mM thymol. As shown in Figure 7B, it was demonstrated that with the increase in the scanning speed from 10 to 200 mV s^−1^, the anodic current of thymol was linearly related to the scanning rate, illustrating that the electrode catalysis of thymol is an adsorption-controlled electrochemical reaction process (Figure 7C). The number of electrons involved in the reaction was calculated from the linear relationship between the oxidation peak potential (*E_p_*) and the logarithm of the scan rate (log *ν*) (Figure 7D), according to the Formula (1) [39]:

(1) $$E_{p}=\frac{2.303RT}{2\left(1-\alpha \right)nf}\log v+K$$

Here, *α* is the transfer coefficient between 0 and 1, *α* is assumed to be 0.5 for a completely irreversible electrode process. *T* is the temperature (298 K), *f* is the Faraday constant (96,500 C mol^−1^), *n* is the number of transferred electrons, and *R* is the gas constant (8.314 J mol^−1^ K^−1^). According to the linear equation: *E_p_* = 0.0483 log *v* + 0.794, the slope shows that thymol had transferred one electron in the oxidation reaction, which is in agreement with previous report [40].


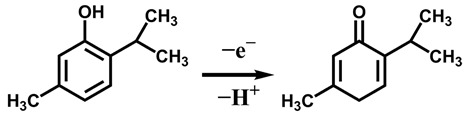


### 2.4. Quantitative Analysis of Thymol

**Figure 8:**
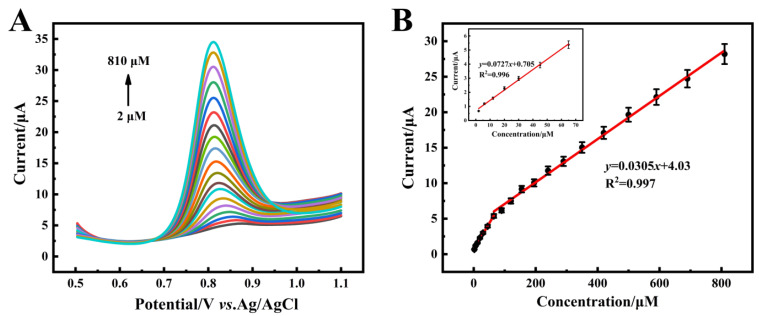
(**A**) DPV of the PtNPs-CPOFs-MWCNTs/GCE in 0.1 M H_2_SO_4_ with different concentrations of thymol from 0.002 mM to 0.810 mM; (**B**) The calibration curve between response currents and thymol concentration.

Under the optimal experimental conditions, the DPV technique was performed to measure the concentrations of thymol in 0.1 M H_2_SO_4,_ and the linear range and detection limit of thymol were studied. The results are displayed in Figure 8. In the range of 2–810 μM, the oxidation peak current increased with the increase in the thymol concentration. The oxidation peak current showed two good linear relationships with the concentration of thymol within the range of 2–65 μM and 65–810 μM. In the range of 2–65 μM, the linear equation is *i*_p_ = 0.0727 *C* + 0.705 (R^2^ = 0.996) and within the range of 65 to 810 μM, it is *i*_p_ = 0.0305 *C* + 4.03 (R^2^ = 0.997). The calculated sensitivities were 72.7 μA mM^−1^ and 30.5 μA mM^−1^, respectively, and the limit of detection (LOD) was estimated to be about 0.2 μM (S/N = 3). Compared with other different methods for thymol determination, the performances of our prepared electrochemical sensor exhibited a superior or comparative linear range and detection limit (Table 1).

### 2.5. Reproducibility, Stability and Selectivity of the Modified Electrode

**Figure 9:**
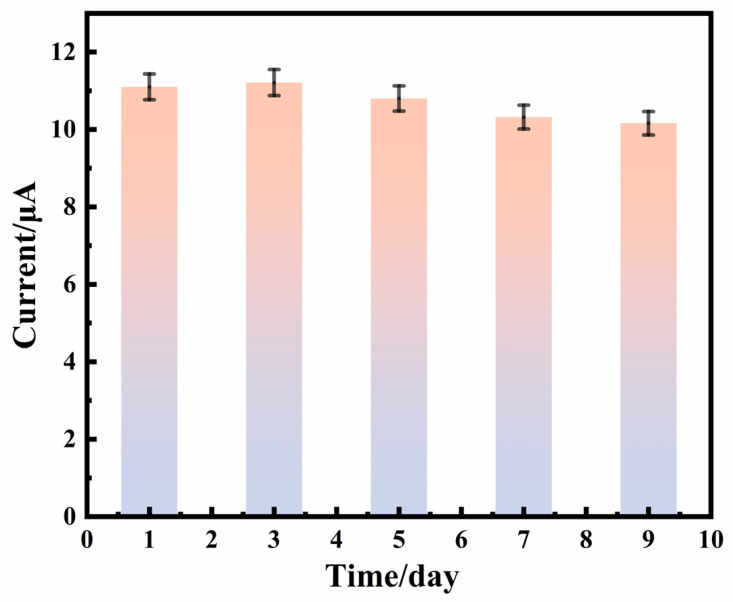
The long-term stability of the thymol sensor.

The reproducibility of the fabricated thymol sensor was tested by detecting the responses to 0.2 mM thymol at five electrodes independently. The results showed that the RSD was 3.67%; meanwhile, the modified electrode was applied to the determination of thymol at 0.2 mM five times with an RSD of 2.79%, suggesting that the prepared thymol electrochemical sensor has good reproducibility. At the same time, the prepared sensor was tested every other day. After 9 days, the current response remained at 91.53% of the initial current, indicating that the sensor had acceptable stability (Figure 9).

**Figure 10:**
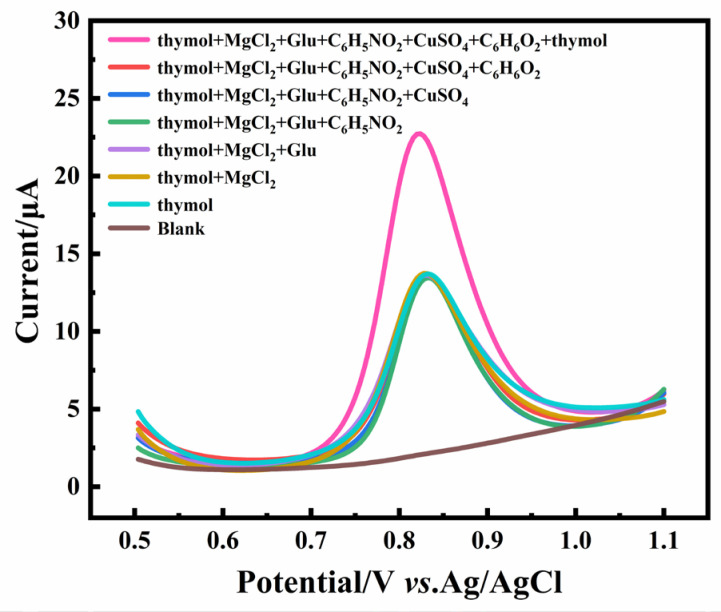
Interference test of the PtNPs-CPOFs-MWCNTs-GCE in the presence of 0.2 mM thymol upon the addition of different interferents, including 0.2 mM MgCl_2_, 0.2 mM Glu, 0.2 mM C_6_H_5_NO_3_, 0.2 mM CuSO_4_, 0.2 mM C_6_H_6_O_2_ and 0.3 mM thymol.

In order to explore whether the designed sensor can be used in real sample testing, the selectivity performance of the sensor was studied, as shown in Figure 10. MgCl_2_, Glu, P-Nitrophenol (C_6_H_5_NO_3_), CuSO_4_ and hydroquinone (C_6_H_6_O_2_) were monitored in the detection system. As shown in Figure 10, there was no obvious interference in the detection of thymol. Therefore, the designed sensor can be used to detect thymol with high selectivity.

### 2.6. Real Sample Analysis

The sensor was applied to analyze the amount of thymol in commercially available thymol powder for children and honey samples in order to assess the feasibility of practical applications. These analyses were performed by the standard addition method, as shown in Table 2. The results showed that the recoveries were 94.45–102.34% and the RSD was less than 5%, indicating that the proposed sensor was suitable for practical application.

## 3. Experimental

### 3.1. Reagents and Materials

Thymol was obtained from Beichen Founder Chemicals (Tianjin, China). MWCNTs, sodium borohydride (NaBH_4_), potassium hexachloroplatinate (K_2_PtCl_6_) and 2,4,6-trihydroxybenzene-1,3,5-tricarbaldehyde were obtained from Aladdin Chemistry Co., Ltd. (Shanghai, China). Tris ((HOCH_2_)_3_CNH_2_) was purchased from Sang Biotech Co., Ltd. (Shanghai, China). Tetrabutylammonium bromide was obtained from Beijing InnoChem Science & Technology Co., Ltd. (Beijing, China). Manganese acetate (Mn(CH_3_COO)_2_·4H_2_O), potassium chloride (KCl), potassium ferricyanide (K_3_[Fe(CN)_6_]), potassium hexacyanoferrate(II) (K_4_[Fe(CN)_6_]), disodium hydrogen phosphate dodecahydrate (Na_2_HPO_4_·12H_2_O) and sodium dihydrogen phosphate dihydrate (NaH_2_PO_4_·2H_2_O) were bought from Tianjin Damao Chemical Reagent Co. (Tianjin, China). All reagents were analytical grade and ultrapure water (≥18.2 MΩ cm) produced by the Millipore system was used for solution preparation.

### 3.2. Methods

CHI760E Electrochemical Workstation (Shanghai Chenhua Instrument Co., Ltd., (Shanghai, China)), a three-electrode system, was used in the experiment with an Ag/AgCl electrode as the reference electrode, a modified electrode as the working electrode and a platinum plate as the counter electrode. The scanning electron microscopy (SEM) images were taken on an S-4800 scanning electron microscope (Hitachi, Tokyo, Japan). The transmission electron microscopy (TEM) images of the nanocomposites were observed using a JEM-2100 microscope (JEOL, Tokyo, Japan). The crystalline materials were analyzed by X-ray diffraction (XRD) using a D/MAX-2500 X-ray diffractometer (Rigaku, Tokyo, Japan). The X-ray photoelectron spectroscopy (XPS) dates were obtained by an ESCALAB 250Xi X-ray photoelectron spectroscope (Thermo Scientific, Waltham, MA, USA). Thermogravimetric analyses (TGA) were carried out on an STD-2960 integration thermal analyzer.

### 3.3. Synthesis of [N(C_4_H_9_)_4_]_3_[MnMo_6_O_18_{(OCH_2_)_3_CNH_2_}_2_] (NH_2_-POM-NH_2_)

NH_2_-POM-NH_2_ was synthesized according to a previous report [35]. A mixture of [N (C_4_H_9_)_4_]_4_[α-Mo_8_O_26_] (0.4000 g), Mn(CH_3_COO)_2_·4H_2_O (0.0750 g) and (HOCH_2_)_3_CNH_2_ (0.0780 g) were dissolved in 25.0 mL acetonitrile, then the mixture was heated at 90 °C for 24 h. The orange solution was cooled to room temperature and exposed the filtrate to ether vapor for 7 days. The orange solid was obtained by filtration and dried in a vacuum at 60 °C. (The synthesis of [N(C_4_H_9_)_4_]_4_[α-Mo_8_O_26_] is described in the Supporting Information).

### 3.4. Synthesis of CPOFs

According to the literature, CPOFs were synthesized by modification [41]. The prepared NH_2_-POM-NH_2_ was used as the monomer and Tp as the other monomer. 50.0 mg NH_2_-POM-NH_2_ and 3.71 mg Tp were dissolved in a mixed solution of 8.0 mL acetonitrile and 8.0 mL 1,4-dioxane by ultrasound, and then 500 μL of acetic acid was added. The mixture was transferred to a Teflon-lined reactor and kept at 100 °C for 2 days. After falling to room temperature, the mixture in the reactor was poured into a centrifugal tube and centrifuged at 10,000 r/min for 10 min to collect the products. The collected solids were soaked in *N*,*N*-dimethylformamide for 24 h and then in acetylene for 48 h. CPOFs were obtained by centrifugal collection and vacuum drying at 60 °C. 

### 3.5. Synthesis of PtNPs-CPOFs-MWCNTs Nanocomposite

Firstly, the PtNPs were fabricated by the chemical reduction method [42]. Briefly, 10 mg K_2_PtCl_6_ was dissolved in 10 mL water and then stirred for 1 h at room temperature. Further, NaBH_4_ solution (0.1 M) was added to the solution drop by drop under vigorous stirring. Following continuous stirring for 2 h, PtNPs were collected by centrifugation (10,000 r/min), washing and drying. Then, to prepare the PtNPs-CPOFs-MWCNTs nanocomposite, PtNPs, CPOFs and MWCNTs were sonicated for 2 h.

### 3.6. Preparation of Different Modified Electrodes

The GCE was first polished with 0.3 and 0.05 μm α-alumina powder successively, followed by successive sonication in water and ethanol several times. The polished electrode was tested in a 0.1 M KCl solution containing 5 mM K_3_[Fe(CN)_6_] for CV. The polished electrode was successful when the redox potential difference was less than 90 mV.

For the fabrication of different modified electrodes, the dispersions of PtNPs, CPOFs and MWCNTs were respectively prepared with 1 mg/mL and ultrasonically treated for 2 h. Then, the mixture of PtNPs and CPOFs with a volume ratio of 5:1 was taken and ultrasonically treated for 2 h to obtain the PtNPs-CPOFs dispersion. Then, 6 μL of PtNPs-CPOFs dispersion solution was used to modify the glassy carbon electrode by the drip coating method to prepare the PtNPs-CPOFs modified electrode labeled as PtNPs-CPOFs/GCE.

The dispersion solution of PtNPs-CPOFs and MWCNTs with a volume ratio of 5:4 was prepared, followed by ultrasound for 2 h to obtain the PtNPs-CPOFs-MWCNTs dispersion; the glassy carbon electrode was modified with 6 μL PtNPs-CPOFs-MWCNTs dispersion by a drip coating method and labeled as PtNPs-CPOFs-MWCNTs/GCE.

## 4. Conclusions

In this study, CPOFs were synthesized by a simple synthesis route and a novel thymol electrochemical sensor based on PtNPs-CPOFs-MWCNTs was successfully constructed. With the excellent oxidation reducibility of CPOFs, the high catalytic activity of PtNPs and the good conductivity of MWCNTs, we obtained a three-in-one sensor that had satisfying selectivity, stability and reproducibility. Under optimal conditions, this electrochemical sensor presented excellent electrochemical properties to thymol with a wide linear range, high sensitivity and low detection limit and has been applied to practical samples. As far as we know, there are few reports on the detection of thymol by the electrochemical method. Our results would break new ground in the field of drug testing.

## Figures and Tables

**Table 1:** Comparison of different methods for thymol determination.

Method	Linear Range	LOD	Reference
HPLC-UV	1.0–80 μg mL^−1^	0.60 μg mL^−1^	[10]
GC-MS/MS	0.050–6.2 μg mL^−1^	14 ng mL^−1^	[11]
Spectrophotometric	0.40–80 μg mL^−1^	0.072 μg mL^−1^	[12]
GCE	0.085–1.3 × 10^−3^ M	79 μM	[13]
MnY/CPE	0.50–28 μM	0.24 μM	[14]
CeO_2_/GN/GCE	0.10–18 μM	0.050 μM	[9]
PtNPs-CPOFs-MWCNTs	2.0–8.1 × 10^2^ μM (0.30–1.2 × 10^2^ μg mL^−1^)	0.20 μM (0.030 μg mL^−1^)	This work

**Table 2:** Determination of thymol in samples based on the proposed sensor (*n* = 5).

Samples	Medicinal Liquid (μM)	Added (μM)	Found (μM)	Recovery (%)	RSD (%)
Honey	–	20	20.15	100.77	2.15
	–	40	37.78	94.45	2.67
	–	60	60.15	100.25	2.85
	–	80	75.70	94.63	2.46
	–	100	98.94	98.94	3.17
Thymol powder	50	150	204.68	102.34	4.08
	50	300	330.62	94.46	3.95
	50	500	549.70	99.95	3.49

## Data Availability

The data that support the findings of this study are available from the corresponding author upon reasonable request.

## Supplementary Materials

The following supporting information can be downloaded at: https://www.mdpi.com/article/10.3390/molecules28083398/s1, Figure S1: The TG of CPOFs (a), PtNPs-CPOFs (b) and PtNPs-CPOFs-MWCNTs (c); Figure S2. The effect of volume ratio of PtNPs and CPOFs on the oxidation current of thymol (0.2 mM); Figure S3. The effect of volume ratio of PtNPs-CPOFs and MWCNTs on the oxidation current of thymol (0.2 mM); Figure S4. The effect of the loading volume of PtNPs-CPOFs-MWCNTs on the oxidation current of thymol (0.2 mM); Figure S5. The effect of the buffer solutions on the oxidation current of thymol (0.2 mM); Table S1: The content of the elements of PtNPs-CPOFs nanocomposite.
