# Supplementary material for: Design and Application of Thymol Electrochemical Sensor Based on the PtNPs-CPOFs-MWCNTs Composite
Source: Molecules. 2023 Apr 12;28(8):3398. doi: 10.3390/molecules28083398 (PMC10143875; doi:10.3390/molecules28083398)
Supplement: Supplementary file 1 [file molecules-28-03398-s001.zip › molecules-2326025-supplementary.pdf]

Supporting Information

Design and Application of Thymol Electrochemical Sensor Based on the PtNPs-CPOFs-MWCNTs Composite

Na Li, Hongyue Zhang, Min Cui, Jujie Ren, Jingru Huang, Bao Sun, Haiyan Zhao and Cong Zhang

Hebei Provincial Key Laboratory of Photoelectric Control on Surface and Interface, School of Sciences, Hebei University of Science and Technology, Shijiazhuang 050018, China

Synthesis of $[N(C_4H_9)_4]_4[\alpha\text{-}Mo_8O_{26}]$

5.00 g Na_2MoO_4 was dissolved in 12 mL of water, then acidified with 5.17 mL of 6 M HCl and stirred for 1-2 min at room temperature. Then 3.34 g of tetrabutylammonium bromide was dissolved in 10 mL of deionized water and added to the flask under vigorous stirring to immediately form a white precipitate. After stirring the mixture for 10 minutes, the precipitate was aspirated and washed with 20 mL water, 20 mL ethanol, 20 mL acetone and 20 mL ether, respectively. The crude product was dissolved in acetonitrile and remained stationary at -10℃ for 24 hours. The clarified, lumpy solids were collected by filtration and dried under vacuum.

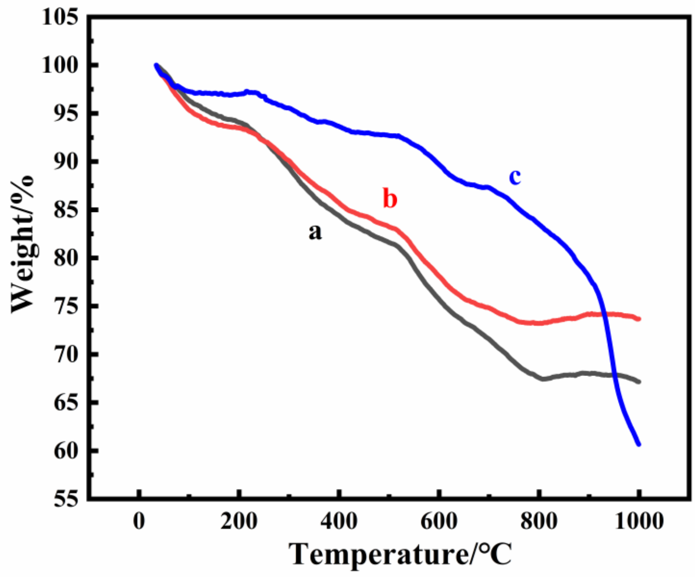

Figure S1. The TG of CPOFs (a), PtNPs-CPOFs (b) and PtNPs-CPOFs-MWCNTs (c).

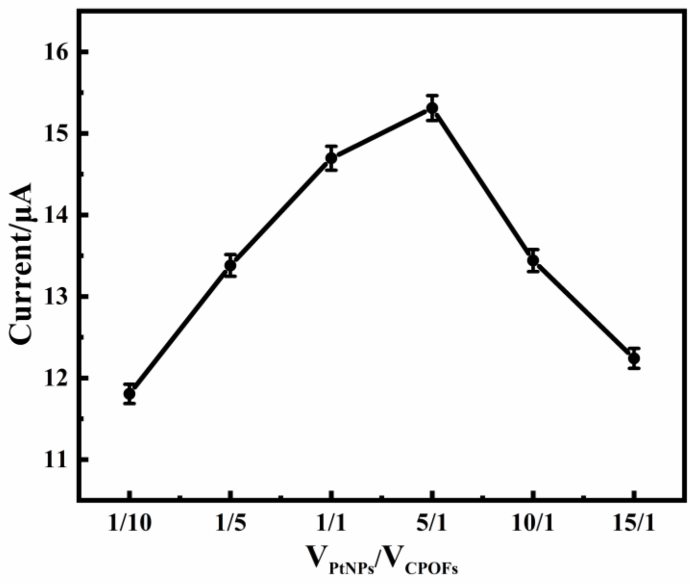

Figure S2. The effect of volume ratio of PtNPs and CPOFs on the oxidation current of thymol (0.2 mM).

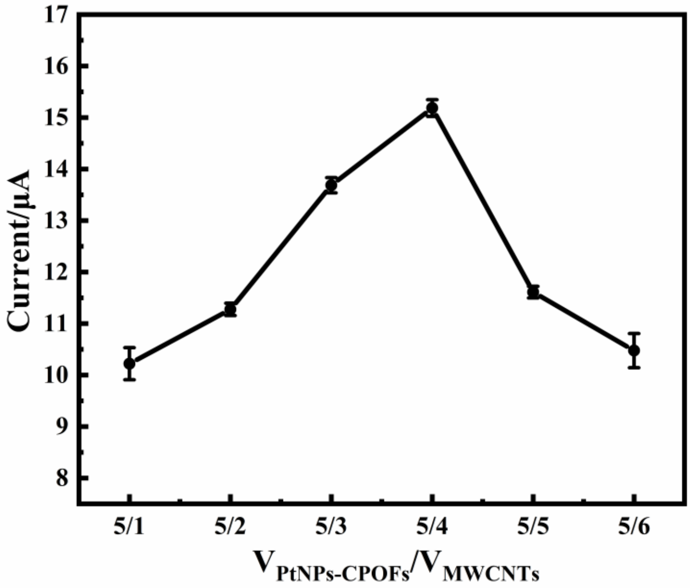

Figure S3. The effect of volume ratio of PtNPs-CPOFs and MWCNTs on the oxidation current of thymol (0.2 mM).

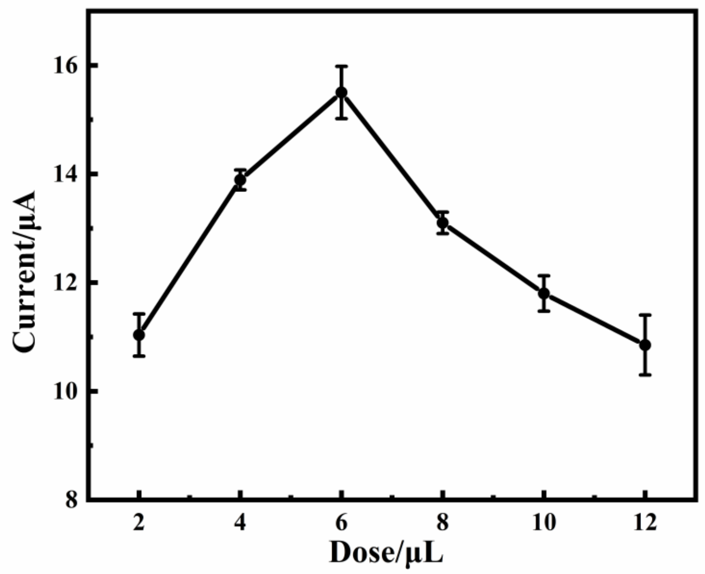

Figure S4. The effect of the loading volume of PtNPs-CPOFs-MWCNTs on the oxidation current of thymol (0.2 mM).

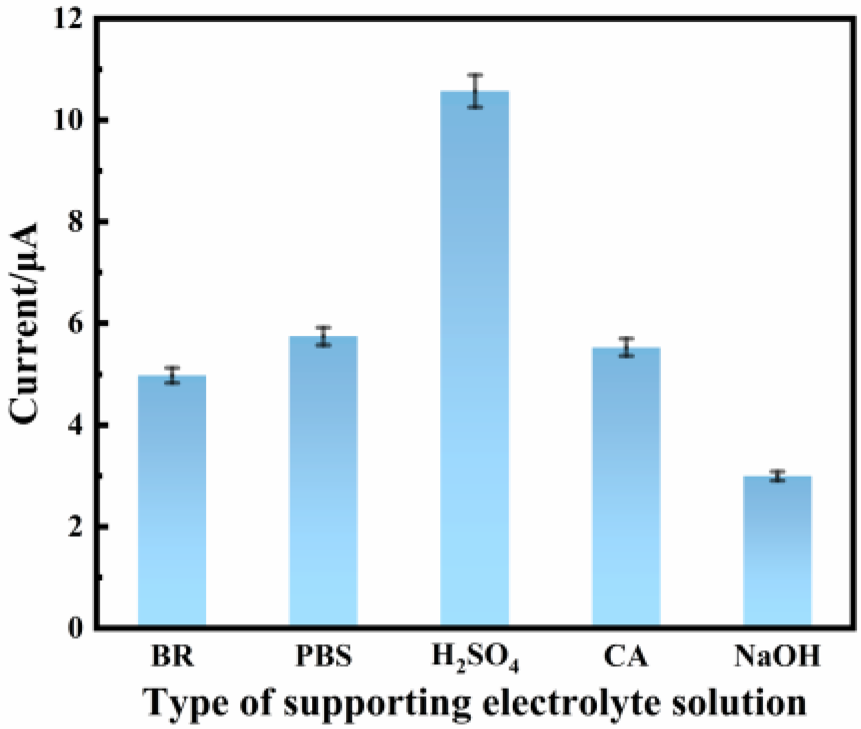

Figure S5. The effect of the supporting electrolytesolutions on the oxidation current of thymol (0.2 mM).

Table S1. The content of the elements of PtNPs-CPOFs nanocomposite.

Element	Element concentration	Strength correction	Weight percentage (%)	Weight percentage (%) Sigma	Atom percentage (%)
C K	57.03	0.4933	36.41	2.03	62.67
N K	0.12	0.1069	0.35	4.42	0.51
O K	34.31	0.4665	23.16	1.31	29.93
Mn K	10.59	0.8441	3.95	0.23	1.49
Mo L	31.80	0.6988	14.33	0.77	3.09
Pt M	61.15	0.8836	21.80	1.10	2.31
